# Synapsis and Meiotic Recombination in Male Chinese Muntjac (*Muntiacus reevesi*)

**DOI:** 10.1371/journal.pone.0019255

**Published:** 2011-04-29

**Authors:** Qingling Yang, Ding Zhang, Mei Leng, Ling Yang, Liangwen Zhong, Howard J. Cooke, Qinghua Shi

**Affiliations:** 1 Hefei National Laboratory for Physical Sciences at Microscale, School of Life Sciences, University of Science and Technology of China, Hefei, China; 2 Department of Biological Sciences, Bengbu Medical Collage, Bengbu, China; 3 MRC Human Genetics Unit and Institute of Genetics and Molecular Medicine, Western General Hospital, Edinburgh, United Kingdom; National Cancer Institute, United States of America

## Abstract

The muntjacs (*Muntiacus*, *Cervidae*) have been extensively studied in terms of chromosomal and karyotypic evolution. However, little is known about their meiotic chromosomes particularly the recombination patterns of homologous chromosomes. We used immunostained surface spreads to visualise synaptonemal complexes (SCs), recombination foci and kinetochores with antibodies against marker proteins. As in other mammals pachytene was the longest stage of meiotic prophase. 39.4% of XY bivalents lacked MLH1 foci compared to less than 0.5% of autosomes. The average number of MLH1 foci per pachytene cell in *M. reevesi* was 29.8. The distribution of MLH1 foci differed from other mammals. On SCs with one focus, the distribution was more even in *M. reevesi* than in other mammals; for SCs that have two or more MLH1 foci, usually there was a larger peak in the sub-centromere region than other regions on SC in *M. reevesi*. Additionally, there was a lower level of interference between foci in *M. reevesi* than in mouse or human. These observations may suggest that the regulation of homologous recombination in *M. reevesi* is slightly different from other mammals and will improve our understanding of the regulation of meiotic recombination, with respect to recombination frequency and position.

## Introduction

Meiotic recombination is essential for segregation of homologous chromosomes at the first meiotic division [1]. In most mammals, including human males, absence or mis-localization of recombination dramatically increases the likelihood of meiotic nondisjunction [2]. At least one recombination event must occur for each pair of homologous chromosomes, with very few exceptions, to ensure accurate segregation of homologs [3].

Most work in mammalian meiosis has focused on humans for reasons of clinical interest or mouse where genetic manipulations are possible. However, little is known about meiotic recombination of homologous chromosomes in other mammals, including muntjacs (*Muntiacus*, *Cervidae*), a well known cytogenetic model due to the wide range of diploid chromosome numbers (i.e. *M. reevesi*, 2n = 46, *M. m. vaginalis*, 2n = 6/7, and *M. crinifrons*, 2n = 8/9), and rapid karyotypic diversification via repeated tandem chromosome fusions [4]. Many studies have focused on chromosomal evolution and nuclear architecture of muntjacs by comparative chromosome banding, chromosome painting and BAC mapping [5]–[10]. Analysis of synaptonemal complexes (SCs) in muntjacs using a surface spreading and silver staining method has been reported [11], but there has been no systematic study on meiotic recombination in this species so far.

Immunofluorescence techniques have been recently developed and widely used to determine genome-wide patterns of recombination on spread preparations of spermatocytes or oocytes from humans and animals [12]–[15]. Antibodies against SYCP3 (a major component of SC lateral elements) were used to visualize SCs (a proteinaceous structure linking homologous chromosomes in prophase of meiosis I). The kinetochores were detected with CREST (calcinosis, Raynaud's phenomenon, esophageal dysfunction, sclerodactyly, telangiectasia) antisera. More importantly, antibodies against the DNA mismatch repair protein MLH1 have been used to identify sites of meiotic recombination on SCs [16], [17]. A major advantage of the approach used in the present study is its applicability to diverse species to establish recombination map. In the present communication, we initiated analyses of recombination and SC configuration in *M. reevesi* spermatocytes which have 46 acrocentric chromosomes, using MLH1 and SYCP3 as markers. Data on meiotic progression, SC length and the distribution of MLH1 foci along SCs were obtained. This allowed us to conduct an initial characterization of genome wide-patterns of recombination and synapsis in male *M. reevesi*.

Studies on various species of mammals demonstrated a wide variation in recombination rates among individuals, populations and species [18]–[21]. Inter-species comparisons are of special interest in view of a long-running discussion about the costs and benefits of recombination [22]. The recombination map of ranked autosomal SCs based on the relative length in a male *M. reevesi* was estimated and compared with that from human males [14] and male mouse [13]. We found that the recombination pattern of *M. reevesi* may be different from other mammals studied so far.

## Results and Discussion

**Figure 1:**
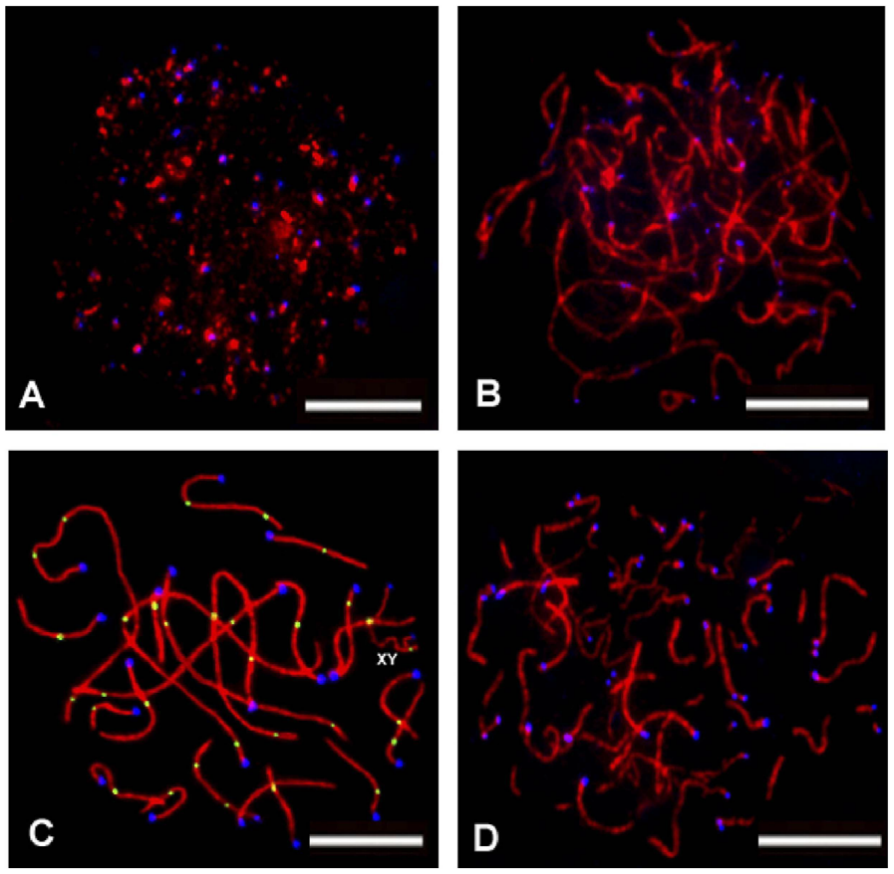
Spermatocytes at different sub-stages of meiotic prophase I in *M. reevesi*. The cells were immunolabeled with antibodies against SYCP3 (red), MLH1 (green) and kinetochores (blue). At leptotene, nuclei displaying as multiple short SYCP3-positive segments and with little association between homologues (A); At zygotene, complete but unpaired SYCP3-positive elements were observed (B); At pachytene, synapsis of homologues was completed with 22 autosomal SCs plus XY pair, MLH1 foci marking recombination sites and 24 centromeric CREST signals were clearly seen, and the appearance of MLH1 recombination sites in sex chromosomes are marked as X and Y (C); At diplotene, SC components disintegrated, recognized by desynapsis of telomeres on some of the bivalents (D). Bar: 10 µm.

The sub-stages of meiotic prophase I in *M. reevesi* were clearly distinguished by immunostaining of SYCP3 on spermatocyte spreads (Figure 1). The frequency of each sub-stage was determined from 167 spermatocytes selected randomly. The frequency of cells at leptotene, zygotene, pachytene and diplotene was 7%, 15%, 59% and 19%, respectively. These data are very similar to that of human males [23], and showed that the pachytene stage is the longest sub-stage.

**Figure 2:**
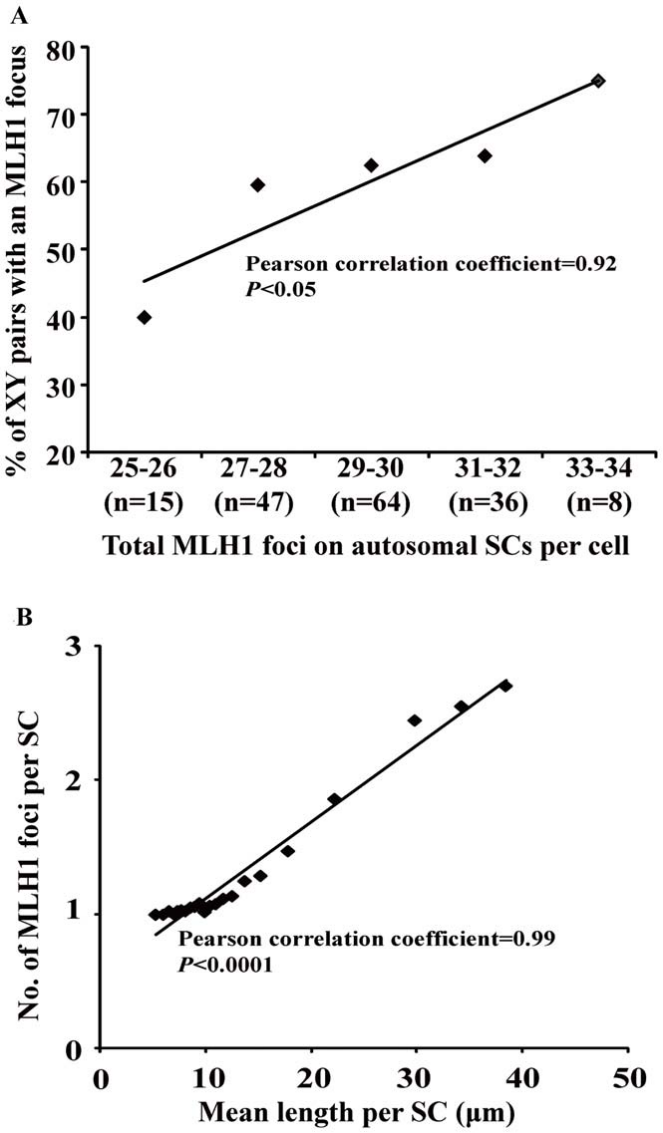
Relationships between autosomal SC length and meiotic recombination frequency in pachytene of male *M. reevesi* (n = 170). (A) Correlation between the frequency of XY pair with an MLH1 focus and total autosomal SC length in a cell. The cells have been divided into four groups based on their total autosomal SC length. (B) A positive correlation between the mean number of MLH1 foci and mean length for individual autosomal SCs (Pearson correlation coefficient = 0.99, *P*<0.0001).

For autosomal SC length, a total of 170 pachytene cells from *M. reevesi* were analysed and autosomal SCs in each cell were ranked based on their relative length (SCs 1–22). The average absolute and relative lengths for *M. reevesi* autosomal SCs were displayed in Table 1. And all these cells were analysed for MLH1 foci in *M. reevesi* (Table 2). For the XY bivalents, 60.6% were observed to have an MLH1 focus, and for autosomal SCs, about 0.5% lacked the MLH1 foci (Table 2), which was similar to those observed in spermatocytes of mouse and humans [16], [17]. The presence of an MLH1 focus in the XY pair was tightly correlated with a frequency of autosomal recombination (Pearson correlation coefficient = 0.92, *P*<0.05) (Figure 2A). Similarly, the percentage of XY pairs showing an MLH1 focus and the mean number of autosomal MLH1 foci per cell have been described to correlate positively in humans [24]. These suggest that the occurrence of recombination on autosomal and sex bivalents is simultaneous.

**Table 1:** Average absolute and relative lengths for *M. reevesi* autosomal SCs.

SC#	Absolute length (µm)		Relative length	
	Mean	SD	Mean	SD
1	38.4	7.3	0.127	0.011
2	34.2	6.6	0.113	0.009
3	29.8	5.5	0.099	0.011
4	22.2	4.7	0.073	0.009
5	17.8	3.8	0.059	0.008
6	15.2	3.2	0.050	0.005
7	13.7	2.8	0.045	0.005
8	12.5	2.5	0.041	0.004
9	11.7	2.1	0.039	0.003
10	11.0	2.0	0.036	0.003
11	10.4	1.8	0.035	0.002
12	9.9	1.6	0.033	0.002
13	9.4	1.5	0.031	0.002
14	9.0	1.4	0.030	0.002
15	8.6	1.3	0.028	0.002
16	8.1	1.2	0.027	0.002
17	7.7	1.1	0.026	0.002
18	7.3	1.1	0.024	0.002
19	6.9	1.1	0.023	0.002
20	6.5	1.0	0.022	0.002
21	6.0	0.9	0.020	0.002
22	5.3	0.8	0.018	0.002
Total	301.5			

# SCs were numbered arbitrarily based on their length in each cell.

**Table 2:** Mean MLH1 foci per cell, frequency of autosomal bivalents with 0–4 MLH1 foci, percentage of cells with an MLH1 focus in XY pair of testicular samples of *M. reevesi*.

No. of cells analysed	MLH1 foci		Mean No. of autosomal SCs when the No. of MLH1 foci is					Cells with an MLH1 focus in XY (%)
	Mean ± SD	Range	0	1	2	3	4	
170	29.8±2.0	25–34	0.1	16.7	3.5	1.5	0.2	60.6

The mean number of MLH1 foci on autosomal SCs per cell was 29.8±2.0, with a range from 25 to 34. A total of 3740 autosomal SCs from 170 pachytene cells were analysed for MLH1 foci distribution based on SC length rank (Table 3). As expected on the basis of previous studies on mouse and human [13], [14], longer SCs contain more MLH1 foci than the shorter ones. Indeed, we found a very strong correlation between the mean MLH1 foci and average length for individual autosomal SCs (Pearson correlation coefficient = 0.99, *P*<0.0001; Figure 2B). Whilst, most SCs showed 1 MLH1 focus, only a few of shortest SCs (ranked 18–22) had no MLH1 foci (Table 3). In mammals at least one crossover event must occur on each SC to ensure proper meiotic segregation of the homologous chromosomes; reduced recombination frequency may thus increase the risk of univalents at metaphase I and of aneuploid gametes [2], [25]. Our results were consistent with previous observations from humans where the shortest autosomes and sex chromosomes are most susceptible to having no recombination foci and thus would be most susceptible to non-disjunction during spermatogenesis [26].

**Table 3:** Distribution of MLH1 foci in each autosomal SC of *M. reevesi* *.

SC#	MLH1 foci		% of individual SC on which the No. of MLH1 foci is				
	Mean	SD	0	1	2	3	4
1	2.64	0.76	0	7.06	31.76	51.18	10.00
2	2.55	0.77	0	8.24	37.06	45.88	8.82
3	2.44	0.69	0	7.06	46.47	42.35	4.71
4	1.88	0.58	0	22.94	66.47	10.00	0
5	1.46	0.51	0	54.71	44.71	0.59	0
6	1.29	0.48	0	71.76	27.06	1.18	0
7	1.24	0.43	0	76.47	23.53	0.00	0
8	1.15	0.39	0	86.47	12.35	1.18	0
9	1.13	0.35	0	87.65	11.76	0.59	0
10	1.08	0.29	0	92.94	6.47	0.59	0
11	1.06	0.24	0	94.12	5.88	0	0
12	1.02	0.15	0	97.65	2.35	0	0
13	1.07	0.26	0	92.94	7.06	0	0
14	1.05	0.22	0	94.71	5.29	0	0
15	1.04	0.20	0	95.88	4.12	0	0
16	1.03	0.17	0	97.06	2.94	0	0
17	1.02	0.15	0	97.65	2.35	0	0
18	1.02	0.17	0.59	97.06	2.35	0	0
19	1.01	0.17	1.18	97.06	1.76	0	0
20	1.02	0.19	0.59	96.47	2.94	0	0
21	1.00	0.15	1.18	97.65	1.18	0	0
22	0.99	0.11	1.18	98.82	0	0	0

*Data from 170 cells.

# SCs were numbered arbitrarily based on their length in each cell.

*M. reevesi* has the same number of chromosomes (22+XY) and a similar average SC length per cell to those of human males (301.5 µm vs. 303.5 µm), and the mean number of MLH1 foci on each chromosome arm in human spermatocytes was comparable to that observed in *M. reevesi* (1.26 vs. 1.32). This phenomenon seems to be shared by males of different mammals [18], a similar mean number of MLH1 foci per autosomal arm (1.21) was observed in mouse spermatocytes (our unpublished data). These data indicate that the MLH1 foci number may be influenced by chromosome structure.

**Figure 3:**
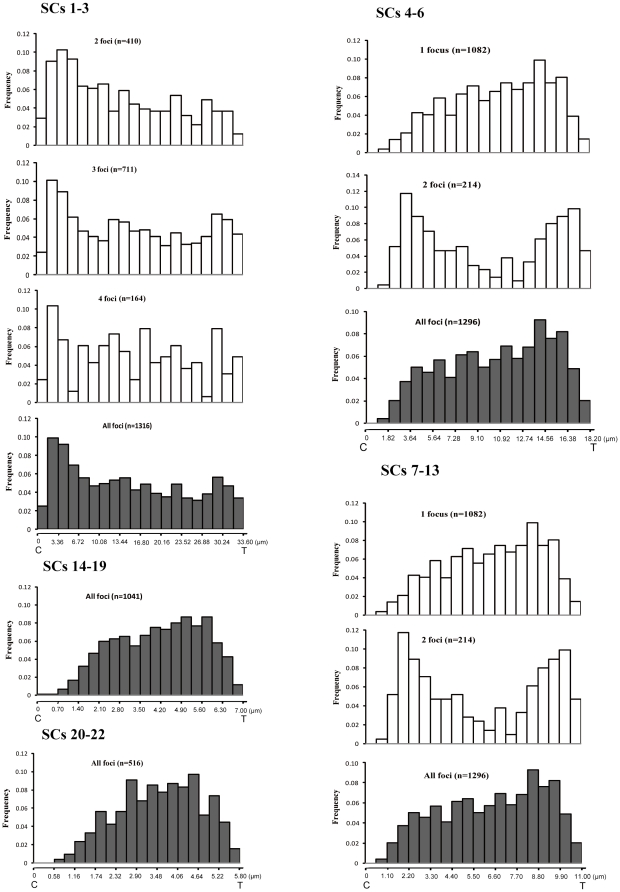
Distribution of MLH1 foci on autosomal SCs from 170 spermatocytes of Chinese muntjac. The SCs have been divided into five groups based on length, SCs 1–3, SCs 4–6, SCs 7–13, SCs 14–19, and SCs 20–22. The X-axis represents the positions on the SCs from the centromeric end (left) to the distal telomere (right), the marks on this axis are separated by 0.05 of the mean absolute SC length for each group. The absolute length (micrometer) scale is shown on the bottom of the last figure for each group. The y-axis indicates the frequency of MLH1 foci in each 0.05 SC interval. For each group, in order from top to bottom, the histograms show the results for SCs with different number MLH1 foci, and the overall frequencies. Data were not displayed when the MLH1 foci<100. n is the number of MLH1 foci analysed.

For all the SC groups, we found that, there is severe repression of MLH1 foci within about 1 µm of centromere (Figure 3). Interference from normal centromeric activity during meiosis or restrictions imposed by the relatively high level of condensation of centromeric heterochromatin may explain the low recombination frequency in sub-centromere region [13], [27], [28]. For the SC 4–22 (group as SCs 4–6, SCs 7–13,SCs 14–19, SCs 20–22, and for the last two groups, each SC mostly has one focus), if there is only one focus on SC, the distribution pattern of the foci depends on the length of the SC. The longer the SC length, the greater the likelihood that the only one focus was near the centromere. When there are two foci on SC, the distribution of the two foci tends to be bimodal, with one major peak near the distal end and the other peak near the centromere. However, for SCs 1–3, When there are two foci on SC, we observed a gradual decrease of frequency from centomere to telomere. The distribution of MLH1 foci shows three small peaks when the SC with 3 or 4 foci. Altogether, for SCs1–3, the highest recombination frequency was not in the region close to telomere ends, which is not only different from SC 4–22 of *M. reevesi* but also different from other mammals where recombination frequency is higher in the region close to telomere [13], [19], [28]–[31]. Previous studies have demonstrated that the recombination patterns are related to the synaptic initiation patterns, and the region with excess of recombination is involved in chromosome alignment and pairing at an earlier stage of meiotic prophase [32]–[34]. Thus, the synaptic initiation pattern may be different from other mammals in *M. reevesi*.

In order to investigate the difference of the recombination patterns among species, we compared the recombination pattern of *M. reevesi* with human males and male mouse [13], [14]. Considering the chromosome morphology may affect the total number of crossovers and the distribution of MLH1 foci on SC, the recombination pattern were compared with the acrocentric chromosomes 13, 14 and 15 in human males. The absolute length of SCs for these chromosomes is about 11 µm each, and most of them bear 2 foci each [14]. *M. reevesi's* SCs 7–13have a similar length to human SCs 13, 14, 15, but have only, on average, 1.2 foci on each SC, which indicated that the distribution of MLH1 foci on SCs is different between the two species. For SCs 20–22 of *M. reevesi*, the absolute length is about 5.8 µm, which is similar to human SCs 21–22 and mouse SCs 6–9, and one recombination focus was observed on most of these chromosomes in the three species, also the distribution of recombination in *M. reevesi* showed non-homogenous pattern when compared with human and mouse which showed a increased frequency in the region close to telomeres [13], [14].

The occurrence of a crossover usually decreases the probability that another will occur nearby. This phenomenon is called crossover interference. Interference may be a factor controlling the upper limit of the recombination [35]. The extent of interference can be determined by the distance between adjacent recombination sites, and it is considered as no interference if the distance is less than 33% of the arm length [31]. In this study, we measured the distance between adjacent MLH1 foci on the same SCs in pachytene cells. The average distance between two neighbouring MLH1 foci was 39.7±18.1% of the arm length for all the autosomal SCs per cell in *M. reevesi* (Table 4), which is smaller than that in all the male mammals studied so far, e.g., human (68%) [14], mouse (70%) [13], cat (47%) [30], shrew (42–55%) [36] and dog (60%) [31]. The average absolute distance between adjacent MLH1 foci was also different among SC groups in *M. reevesi* (Table 4). The minimum inter-focus distance on SC of *M. reevesi* was 0.3 µm in the present study (Table 4), which is shorter than male mouse (0.8 µm) [28] and human males (about 1 µm) [14]. These observations indicate that the strength of interference is weaker in *M. reevesi*, which possibly explains the more random distribution of MLH1 foci along SCs in *M. reevesi* than in other mammals. The distance of 0.31 µm may be the minimum that is physically possible to accommodate neighboring exchange events.

**Table 4:** Mean distances between two adjacent MLH1 foci in SC groups of different length.

SC groups	No. of SCs observed	Mean absolute distance between adjacent foci (µm)		Mean relative distance between adjacent foci (% of SC length)	
		Average ± SD	Range	Average ± SD	Range
SCs 1–3	798	11.6±5.4	0.3–34.6	34.1±15.3^a^	1.1–87.6
SCs 4–6	284	10.1±4.7	1.7–27.1	50.0±19.0^a^	8.6–89.9
SCs 7–13	107	6.8±2.6	0.4–17.1	53.3±15.7^a^	22.0–88.9
SCs 14–19	24	3.5±2.0	1.3–8.2	43.1±22.0^a^	14.8–83.8
SCs 20–22	5	2.3±0.9	1.3–3.3	40.4±19.0^b^	24.1–65.0
Mean				39.7±18.1	

a Significant different , one-way ANOVA, *P*<0.01.

b Not included in one-way ANOVA test because <10 observations.

Altogether, the distribution pattern in *M. reevesi* was a little different from other mammals studied so far, this may suggests that in turn the regulation mechanism of homologous recombination in *M. reevesi* is slightly different from other mammals. The molecular basis of this difference remains unknown.

This is the first attempt, using a simple cytological approach, to study the meiotic events in male *M. reevesi*. The muntjacs have a very wide distribution of chromosome numbers (i.e. from 2n = 46 in *M. reevesi*, 2n = 8/9 in *M. crinifrons* and 2n = 6/7 in *M. m. vaginalis*), which makes them a paradigm for the studies of chromosomal and karyotypic evolution. The radical decrease in the chromosome number in *M. crinifrons* and *M. m. vaginalis* was thought to result from repeated tandem fusions of chromosomes in primal *M. reevesi* [4]. Our analysis provides estimates of several important characteristics of the *M. reevesi* genome: meiotic progression, the relationship between SC length and recombination frequencies, the recombination distribution pattern on autosomal and XY bivalents and crossover interference. Our data presented here provide the basis for future comparative analysis of recombination characteristics among muntjac species with widely differing karyotypes. The difference in the recombination pattern of *M. reevesi* from other mammals indicates that the regulation mechanism underlying homologous recombination is different between species and also highlights the need for studies on more species to understand the evolution of the regulation of homologous recombination.

## Materials and Methods

### *M. reevesi*

Testicular tissue was obtained from a wild male M. reevesi, which was from wild animal's refuge area of Anhui Province, China. The collection and using M. reevesi tissue were under the approval of the Institutional Review Board at University of Science and Technology of China, the approval ID: USTCAU201000004.

### Immunofluorescence

SC spreads of spermatocytes were prepared from an animal using the technique described by Judis *et al.* (2004). To analyse synapsis and recombination, rabbit anti-SYCP3 (1∶3000, gifted from Christa Heyting), human anti-CREST (1∶1000, Immunovision) and mouse anti-MLH1 (1∶ 60, BD Pharmingen biosciences) antibodies were added to the slides and incubated overnight at 37°C. On the following day, the secondary antibody mixture was applied: Alexa 555 donkey anti-rabbit (1∶250, Molecular Probes), Alexa 488 goat anti-mouse (1∶100, Molecular Probes) and 1-amino-4-methylcoumarin-3-acetic acid (AMCA) donkey anti-human (1∶200, Jackson Immunoresearch). Slides were incubated at 37°C for 1.5 h, then 20 µl of vectashield (Vector Laboratories) was applied, and a coverslip was sealed in place.

### Data analysis and establish recombination map

An epifluorescence microscope OLYMPUS BX61 and Image Pro-Plus version 5.1 software (Media Cybernetics Inc., Bethesda, MD, USA) were used for cell analysis and image, and we measured the absolute length of each autosomal SC by using Image Pro-Plus 5.1 software. To determine the relative length of each SC, the absolute length for each SC was divided by the total absolute length of all autosomal SCs in the cell. All autosomal SCs were ranked in the order of decreasing relative length (SCs 1–22). To determine the distribution of MLH1 on SCs we measured the distance of each MLH1 focus to the centromere along the SCs. The distribution of MLH1 foci on each autosomal SC were then obtained by averaging the number of foci on the SC among the cells analysed. Previous studies indicated that the absolute length of SC correlates with the length of mitotic chromosome for most chromosomes [11], [12]. Based on this finding, we combined the SCs with similar absolute length in the following groups: SCs 1–3, SCs 4–6, SCs 7–13, SCs 14–19 and SCs 20–22. By analysing the data in groups of chromosomes we smooth any effect of mistaking SC's of similar size. We measured the length of each autosomal SC and the distance from each MLH1 focus to the centromere. Then, the long arm of each SC was divided into 20 intervals of equal length and the frequencies of MLH1 foci in each interval were plotted (Figure 3). Data were not displayed when the MLH1 foci were fewer than 100.

### Statistical analysis

Statistical analysis was performed using SPSS 13.0 software. Pearson's correlation test was used to identify a relationship between the average length of each SC and the mean number of MLH1 foci on that SC as well as the frequency of XY pairs with an MLH1 focus and the total MLH1 foci on autosomal SCs in a cell.
